# The association between pain diagram area, fear-avoidance beliefs, and pain catastrophising

**DOI:** 10.1186/2045-709X-22-5

**Published:** 2014-01-20

**Authors:** Bruce F Walker, Christine D Losco, Anthony Armson, Amanda Meyer, Norman J Stomski

**Affiliations:** 1 School of Health Professions, Murdoch University, Murdoch, Australia

## Abstract

**Background:**

The development of clinical practice guidelines for managing spinal pain have been informed by a biopsychosocial framework which acknowledges that pain arises from a combination of psychosocial and biomechanical factors. There is an extensive body of evidence that has associated various psychosocial factors with an increased risk of experiencing persistent pain. Clinicians require instruments that are brief, easy to administer and score, and capable of validly identifying psychosocial factors. The pain diagram is potentially such an instrument. The aim of our study was to examine the association between pain diagram area and psychosocial factors.

**Methods:**

183 adults, aged 20–85, with spinal pain were recruited. We administered a demographic checklist; pain diagram; 11-point Numerical Rating Scale assessing pain intensity; Pain Catastrophising Scale (PCS); MOS 36 Item Short Form Health Survey (SF-36); and the Fear Avoidance Beliefs Questionnaire (FABQ). Open source software, GIMP, was used to calculate the total pixilation area on each pain diagram. Linear regression was used to examine the relationship between pain diagram area and the following variables: age; gender; pain intensity; PCS total score; FABQ-Work scale score; FABQ-Activity scale score; and SF-36 Mental Health scale score.

**Results:**

There were no significant associations between pain diagram area and any of the clinical variables.

**Conclusion:**

Our findings showed that that pain diagram area was not a valid measure to identify psychosocial factors. Several limitations constrained our results and further studies are warranted to establish if pain diagram area can be used assess psychosocial factors.

## Introduction

The 2010 Global Burden of Disease study reported that musculoskeletal disorders ranked worldwide as the second leading cause of disability [1]. In considering individual conditions, the two most prevalent types of spinal pain, low back pain and neck pain, were respectively the leading, and fourth leading, source of disability-adjusted life years [1]. The extent of this problem leads to considerable socioeconomic burden in both direct medical costs and indirect costs [2].

The development of clinical practice guidelines for managing spinal pain have been informed by a biopsychosocial framework which acknowledges that pain arises from a combination of psychosocial and biomechanical factors [3-6]. About 80% of people seeking care for spinal pain will have non-specific spinal pain, for which assigning diagnostic labels is not recommended, and the approach to management depends on the clinician’s and patient’s preferences [6,7].

Psychosocial factors encompass social and socio-occupational factors, psychological factors, cognitive and behavioural factors [8]. There is an extensive body of evidence that has associated various psychosocial factors with an increased risk of developing persistent pain, these include pain catastrophising; fear avoidance beliefs; anxiety; and depression [9-11].

Clinicians require instruments that are brief, easy to administer and score, and capable of validly identifying such psychosocial factors [10]. This information potentially enables clinicians to identify patients at risk of developing persistent pain and subsequently use targeted interventions that address psychosocial factors [12-14].

**Figure 1:**
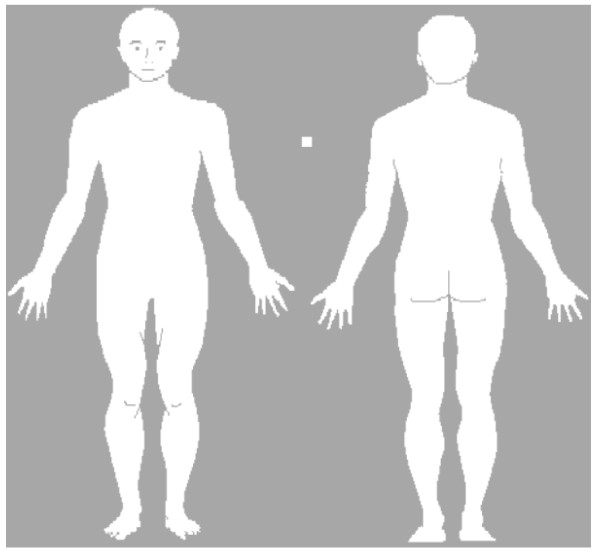
Pain diagram.

The pain diagram is one instrument that has been used to assess psychosocial factors [15]. It comprises front and back outlines of a body on which patients may mark areas that correspond to the distribution of pain (Figure 1). Several scoring methods have been used to identify psychosocial factors, including the Ransford Scoring System [16]; Modified Ransford Scoring System [17]; Pain Sites Scoring System [18]; and Body Map Scoring System [18]. However, none of these systems have been found to be valid measures [19].

One previous study has shown that the quantification of shaded pain diagram area can be reliably assessed and used to predict outcomes such as work absence and occupational disability [20]. Such findings have led to the inference that pain diagram area may be associated with psychosocial factors as these factors affect work absence and occupational disability. Scant research appears to have examined the relationship between pain diagram area and psychosocial factors that influence spinal pain. Therefore, the aim of our study was to examine the association between marked pain diagram area and two important psychosocial factors, pain catastrophising and fear-avoidance beliefs.

## Methods

### Study design

In this manuscript, we detail a secondary analysis which examined the relationship between the area marked on a pain diagram and psychosocial variables. The primary analysis examined adverse events resulting from chiropractic treatment and this has been published elsewhere [21,22]. The trial was registered with the Australian New Zealand Clinical Trials Registry (ACTRN12611000542998) and the protocol has been published [23]. All data were collected over one month between August and September 2011 at Murdoch University, Western Australia.

### Recruitment and eligibility criteria

Newspaper advertisements were used to recruit participants from the local community. All participants were adults; English literate; experienced current spinal pain (neck, mid-back, or low back pain) of at least one week duration; and scored at least three out of a maximum 10 on the Numerical Rating Scale (NRS) for pain [24], and 12 out of a maximum of 40 on the Functional Rating Index (FRI) [25].

We excluded participants who indicated they would be unwilling to tolerate any intervention possibly delivered in usual chiropractic care including: manipulative therapy; mobilisation; traction; massage; ultrasound; and physical modalities. Additional exclusion criteria were: spinal pain related to cancer or infection; spinal fracture; spondyloarthropathy; known osteoporosis; progressive upper or lower limb weakness; symptoms of cauda equina syndrome or other significant neurological condition; recent disc herniation; known severe cardiovascular disease; uncontrolled hypertension; cognitive impairment; blood coagulation disorder; previous spinal surgery in the past year; previous history of stroke or transient ischaemic attacks; pacemaker or other electrical device implanted; substance abuse issues; pregnancy; or a current compensation claim.

### Baseline assessment

Full details about the administration of baseline and outcome measures have been reported elsewhere and the following section only contains details germane to this study [21,22]. At baseline we administered a demographic checklist; pain diagram [15]; 11-point Numerical Rating Scale assessing pain intensity [24]; Pain Catastrophising Scale (PCS) [26]; Medical Outcomes Study 36 Item Short Form Health Survey (SF-36) [27]; and the Fear Avoidance Beliefs Questionnaire (FABQ) [28]. We used the posterior and anterior views of the human body seen on the pain diagram. Participants were asked to shade areas on the pain diagrams corresponding to where they were currently experiencing pain or discomfort. The SF-36 is a well validated and extensively used measure of general health status [27]. The NRS contains an 11-point scale ranging from 0 (no pain) to 10 (worst pain imaginable). It is a valid, reliable and responsive measure of pain intensity [24]. The PCS assesses different dimensions of negative thoughts related to pain: rumination, magnification, and helplessness. The PCS has been shown to be a reliable and valid measure that predicts clinical outcomes [26]. The FABQ evaluates patient beliefs about the effect of physical activity and work on their experience of pain. The FABQ has good test-retest reliability, and predictive validity for future episodes of pain and clinical outcome [28].

### Instrument of measurement

We used open source software, GIMP, to calculate the total marked pixilation area on each pain diagram [29]. It should be noted that only the pixels marked on the pain diagrams were counted. All pain diagrams were scanned and stored as JPEG files using RICOH Aficio MP4001 model scanner. GIMP’s automatic particle analysis function was then used to calculate the total number of pixels marked on each pain diagram [29]. A five step process using GIMP (version 2.6) was instituted. This included standardization of (i) size and (ii) colour, then (iii) determination of range of interest, (iv) defining different qualities of pixilation to a dichotomous choice. Pixel analysis with histogram function and quantification has been validated for objective quantification by Schönberger [30].

### Ethics

Ethics approval was obtained from Murdoch University’s Human Research Committee (2011/109).

### Data analysis

Data were analysed in SPSS v.21. Linear regression was used to examine the relationship between pain diagram area and the following variables: age; gender; pain intensity; PCS total score; FABQ-Work scale score; FABQ-Activity scale score; and SF-36 Mental Health scale score. Age, gender, and pain intensity were entered as covariates as they had been identified as effect modifiers in previous studies [19]. In addition, we constructed another linear regression model to examine the relationship between pain intensity and the following variables: PCS total score; FABQ-Work scale score; and FABQ-Activity scale score.

## Results

### Demographic and clinical characteristics

Baseline measures were completed by 183 participants. Table 1 displays the participants’ demographic and clinical characteristics. Most participants were male, and the majority had middle to high income levels and had completed secondary school.

**Table 1:** Participant baseline demographics and clinical characteristics

Demographics*	**No (%)**
Mean age in years, (SD)	55 (14.5)
Women	67 (36.6%)
Low income	55 (30.9%)
Middle/High income	123 (69.1%)
Did not complete secondary school	50 (27.4%)
Currently smoke	50 (27.4%)
Moderate/Heavy alcohol consumption	67 (36.6%)
**Clinical characteristics**	**Mean (SD)**
Numerical Rating Scale	5.0 (1.7)
Pain Diagram Pixilation Area	129388.6 (6479.2)
Pain Catastrophising Scale	15.1 (10.6)
Fear Avoidance Beliefs Questionnaire-Activity	13.9 (5.7)
Fear Avoidance Beliefs Questionnaire-Work	15.7 (10.9)
SF-36 Mental Health Component Score	73.1 (17.9)

*Figures are number (percentage) unless stated otherwise.

Almost all patients (98%) had experienced spinal pain for more than three months. Three quarters (74%) had experienced spinal pain for more than five years. The vast majority (94%) stated it had been more than one year since last experiencing a four week pain free period, and over two thirds (69%) indicated it had been more than one year since their last one week pain free period.

### Association of pain diagram with demographic and clinical characteristics

Table 2 displays the results of the linear regression analyses. There were no significant associations between marked pain diagram area and any of the demographic or clinical variables.

**Table 2:** Associations between pain diagram area and demographic and clinical characteristics

	**β**	**95% CI**
Gender	0.02	-1959.30–2401.12
Age	0.04	-56.91–88.59
Baseline pain intensity	0.07	-413.48–916.21
Pain Catastrophising Scale	-0.12	-192.59–47.12
Fear Avoidance Beliefs Questionnaire - Work	0.08	-59.45–147.39
Fear Avoidance Beliefs Questionnaire - Activity	0.06	-132.09–266.68
SF-36 Mental Health Component Score	-0.09	-92.81–30.39

### Associations between pain intensity and psychosocial factors

Table 3 displays the results of the linear regression analyses. Pain intensity was significantly associated with pain catastrophising and fear-avoidance work beliefs, but not significantly associated with fear-avoidance activity beliefs.

**Table 3:** Associations between pain intensity and pain catastrophising and fear-avoidance beliefs

	**β**	**95% CI**
Pain Catastrophising Scale	0.05	0.03–0.07
Fear Avoidance Beliefs Questionnaire - Work	0.03	0.01–0.06
Fear Avoidance Beliefs Questionnaire - Activity	0.01	-0.04–0.05

## Discussion

To our knowledge, this was the first study to examine the relationship between pain diagram area, calculated by the use of computer software, and psychosocial factors. Although pain intensity was significantly associated with both fear-avoidance work beliefs and pain catastrophising, we observed no relationship between pain diagram area and pain catastrophising, fear-avoidance beliefs, and psychological health (assessed by the SF-36 Mental Health component score. Previous studies using different scoring systems to the system used in this study also found that pain diagrams could not validly identify the presence of psychosocial factors [15,17,18,31-40].

One previous study had demonstrated a significant association between low SF-36 Mental Health component scores and pain diagrams that exhibited non-organic features based on the scoring system developed by Mann et al. [41]. This system classifies a pain diagram as nonorganic when the following features are observed: an excessive number of pain markings; a wide distribution of marks over many anatomic regions; marks outside the silhouette; and a disregard of instructions on what symbols to use. In cases where non-organic pain diagrams were identified, the odds ratio of obtaining a non-organic pain drawing was 22 when the Mental Health scale score was more than two standard deviations below the Danish norm. That finding suggests that the non-organic feature scoring system, in comparison to pain diagram area, is a superior method to identify psychological health through the use of pain diagrams. However, before this method could be confidently recommended, subsequent studies would be required to confirm the validity of using non-organic drawings to assess psychological distress.

Several limitations constrained the findings of this study. We did not assess test-retest reliability and are uncertain if this influenced our results. Another issue involves the participants using different types of ball point pens to fill out the pain diagram, and although these do not vary in a substantial way it is unclear as to what extent different pen tip widths may have affected the calculation of pain diagram area. Finally, the reliability of GIMP software has not been fully established, and so we are uncertain if this may have biased our results.

## Conclusion

Our findings indicated that pixilation area on pain diagrams could not be used to identify psychosocial factors, but the study limitations raises uncertainty about the results and further studies are warranted. However, the procedure involved with calculating pixilation area was laborious and it is doubtful whether clinicians would be prepared to use the procedure even if it could be used assess psychosocial factors. Hence, we suggest that further research in this area is unlikely to be of great value, particularly as clinicians can readily identify patients in need of psychosocial interventions through the use of brief, validated instruments such as the Startback Screening Tool [42].

## Competing interests

BW and NS are on the editorial team of Chiropractic and Manual Therapies but had no involvement in the editorial process of this manuscript.

## Authors’ contributions

BW and NS contributed to the conception and design of the study. BW, NS and CL assisted with acquisition of data. All authors contributed to analysis and interpretation of data, drafting the manuscript, and have given final approval of the version to be published.
